# Study of the Relation Between the Reynolds Number and the Formation of Au and Ag Nanostructures by Flow-Driven Surface Modification in Microfluidic Reactors

**DOI:** 10.3390/mi17040470

**Published:** 2026-04-14

**Authors:** Oscar Perez-Landeros, Alan Garcia-Gallegos, David Mateos-Anzaldo, Roumen Nedev, Judith Paz-Delgadillo, Mariela Dominguez-Osuna, Evelyn Magaña-Leyva, Ricardo Salinas-Martinez, Mario Curiel-Alvarez

**Affiliations:** Instituto de Ingeniería, Universidad Autónoma de Baja California, Blvd. Benito Juárez s/n, Mexicali 21280, Mexico

**Keywords:** microfluidics, microreactors, nanostructures, plasmonic surfaces, galvanic displacement, flow-driven surface modification, additive manufacturing

## Abstract

Microfluidics enables spatially controlled nanostructure synthesis by coupling confined flows with surface reactions. In this work, we study how geometry-induced laminar microenvironments govern the in situ formation of Au and Ag nanostructures inside 3D-printed microfluidic reactors. Proof-of-concept fish-scale valves were fabricated by masked stereolithography in three architectures designed to define three recurring zones in the microreactor, inside the fish-scales (zone 1), between the fish-scales (zone 2), and along the rows of fish-scales (zone 3). A Cu thin film was deposited on the inner walls of the channel to serve as the sacrificial surface for galvanic replacement using AgNO_3_ or HAuCl_4_. Distinct 0D, 1D, and 2D nanostructures were simultaneously obtained in a zone-dependent manner across the valves, including nanoparticle and nanopore-rich regions, nanowires, nanoflakes and clustered 2D features. COMSOL simulations were used to solve the Navier–Stokes equation and extract specific-zone flow descriptors, including Reynolds number, velocity, and wall shear stress, and relate them to the nanostructure morphologies observed by SEM. The flow throughout the devices is strongly laminar, with local Reynolds numbers up to 0.04, exhibiting systematic spatial gradients imposed by the valve geometry. These results provide a design-guided route to tune nanostructure morphology through microchannel architecture under constant global operating conditions.

## 1. Introduction

Over the last decade, microfluidics has seen its application in the synthesis of nanoparticles of noble metals [1,2,3], semiconductors [4], and metal colloids [5]. In particular, metallic and semiconductor nanostructures have been extensively studied due to their interaction with light, which can be used in various applications such as sensing, molecule capture, and degradation [6,7,8]. The fabrication of microfluidic chips includes an extensive study of the materials’ selection, and the substrate of choice as well; the most used materials in the fabrication of microchannels are polydimethylsiloxane (PDMS) [9], polymethyl methacrylate (PMMA), and polyvinyl chloride (PVC). Microfluidic devices expand the possibility of working with small sample volumes and enable portable devices for on-site testing [10]. Additionally, microfluidics has been used in even low-cost techniques by fabricating paper-based devices for the in situ synthesis of gold (Au) nanoparticles for the detection of biomarkers for a medical approach [11]. Likewise, a microfluidic chip has been reported that is capable of analyzing the biomechanical properties of blood for monitoring early-stage diseases [12]. Additive manufacturing (AM) is the process of producing a 3D object layer by layer, commonly using polymer filaments such as polylactic acid (PLA) or acrylonitrile butadiene styrene (ABS) [13]. Vat photopolymerization (VP) is a subcategory of AM and has drawn attention due to its high printing resolution, easy production of complex geometries, and low energy and material consumption [14]. VP consists of a container that contains a photosensitive resin, which solidifies when exposed to ultraviolet light for a specific exposure time and intensity, obtaining a layer-by-layer 3D object with high resolution [15]. This method is an efficient way for rapid prototyping of rigid and flexible microfluidic devices in a variety of polymers.

These printed devices are not only useful as fluidic platforms but also enable the in situ synthesis of metallic nanoparticles within their internal microchannels. In this manuscript, a flow-driven, in situ transformation of a Cu-coated microchannel surface by galvanic replacement is described. Under constant global operating conditions, the fish-scale geometry creates distinct microenvironments (zones) that impose different dynamics of the local transport and reactions, yielding zone-dependent nanostructures along the reactor. In this context, the synthesis of metallic nanoparticles (NPs) was achieved through galvanic replacement reactions. The dominant variables in this method of synthesis, coupled with our microfluidic approach, are the characteristic length of the microchannel [16], the concentration of the solution [17], the flow rate [18], the pressure [19], and the reaction time [20]. In microchannel regimes, the flow parameters are characterized by laminar flow behavior with a Reynolds number (*Re*) less than 2000, making the modeling easier by using the Navier–Stokes equation compared to the chaotic behavior of turbulent regimes [21]. In this work, the *Re* was studied, because it specifies the ratio between the effect of the inertial force vs. the viscous force in the fluid flow. In microfluidic reactors, it is possible to obtain highly laminar flow, which, in terms of *Re*, corresponds to values ≤ 1. Therefore, the viscous force dominates the fluid [22]. Although vortices are not expected in a laminar regime, it is possible to achieve localized vortices. Reports have shown that tuning the geometry of microchannels can create microvortices, which serve as traps for particles and cells [23]. Noble metallic nanostructures are widely used in different areas, such as sensors for medical purposes [24,25], tribo nanogenerators for energy harvesting [26], and photocatalysis [27], among others. In this work, we estimate parameters that are not directly measurable, such as *Re*, shear stress, and flow velocity, using COMSOL simulations to relate them to the formation of metallic nanostructures by galvanic displacement in a laminar flow condition and to understand the role of the *Re* in the synthesis of these nanostructures. Although microfluidic and additively manufactured platforms have been increasingly explored for metallic nanostructure synthesis, most studies rely on simple channel configurations and tune particle formation mainly through global operating variables such as flow rate, residence time, or reagent ratios, leaving the role of geometry-defined local hydrodynamic microenvironments in 3D-printed reactors still insufficiently understood. In this work, we investigate how fish-scale microchannel architectures generate distinct laminar microenvironments that direct the zone-dependent in situ formation of Ag and Au nanostructures by galvanic replacement on Cu-coated surfaces. The novelty of this study lies in combining MSLA-fabricated microreactors, spatially recurring reaction zones, and COMSOL-derived local descriptors, including Reynolds number, velocity, and wall shear stress, to establish a structure–flow correlation under constant global operating conditions.

## 2. Materials and Methods

### 2.1. Chemical Reagents

The materials used for the fabrication of silver and gold nanostructures (NSs) include 99.999% pure copper (Cu, Ted Pella, Inc., Redding, CA, USA) for thermal evaporation, 99.0% pure silver nitrate (AgNO_3_, Sigma-Aldrich, Inc., St. Louis, MO, USA) and 99.9% pure tetrachloroauric acid (HAuCl_4_, Sigma-Aldrich, Inc., St. Louis, MO, USA) for galvanic replacement. A Flow EZ microfluidic flow controller (Fluigent, Le Kremlin-Bicêtre, France) was used to drive and control reagent flow. The microfluidic reactors were made of transparent photosensitive resin.

### 2.2. Characterization Techniques

The fish-scale valve areas, chemical composition, and morphology of the prepared NSs were studied by scanning electron microscopy (SEM) and energy dispersive X-ray spectroscopy (EDS) using a Lyra FE-SEM (Tescan, Brno, Czech Republic) with a built-in Bruker EDS detector (Billerica, MA, USA).

### 2.3. Design of the Microfluidic Fish-Scale Valve

**Figure 1:**
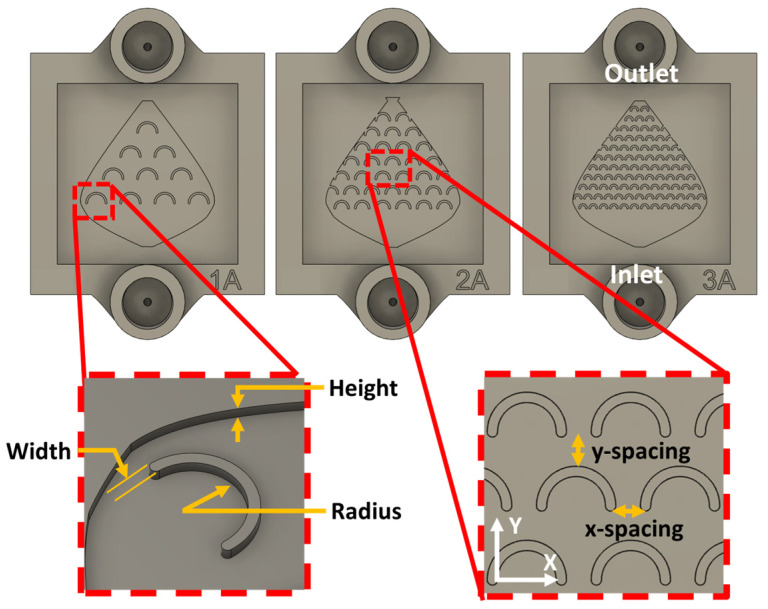
Definition of the zones of characteristic features in the 1A, 2A, and 3A fish valves.

Microfluidic chips were designed, fabricated, and studied in order to identify the key parameters governing flow-driven in situ nanostructure synthesis inside the microchannel. The microfluidic chips, also referred to as fish-scale valves, were printed using the masked stereolithography (MSLA) technique on an Anycubic Photon Mono 4K 3D printer (Shenzhen, China), which operates with a 405 nm light source, with a nominal resolution of 35 µm (X and Y axes) and 10 µm (Z axis), using Anycubic High Clear resin (Shenzhen, China). The height of the microfluidic device was chosen to maintain a laminar regime and to locally alter the flow regime at specific zones within the microfluidic chip, aiming to achieve higher velocities and vortex spots. We fabricated three different versions of the microfluidic fish-scale valve chips, each with a channel height of 200 µm. These valves contain an internal C-shaped scale-like microstructure that generates constant vortex spots and shifts the flow regime. The inner microstructures (referred to as fish-scales) are uniformly distributed along the active surface of the devices, with 10, 33, and 100 fish-scales for devices 1A, 2A, and 3A, respectively (Figure 1). The fish-scales in devices 1A, 2A, and 3A are separated transversely to the flow direction (X axis) by 1.3 mm, 500 µm, and 150 µm, respectively, and along the flow direction (Y axis) by 1.3 mm, 600 µm, and 450 µm, respectively. The C-shaped scale-like structures have a height and width of 200 µm for all devices and a radius of 900 µm, 600 µm, and 250 µm for the 1A, 2A, and 3A devices, respectively. Table 1 summarizes the geometrical parameters of the three device types, and Figure 1 shows the spatial position of the dimensions. The total channel length and width in all three devices are 16 mm and 14 mm, respectively.

### 2.4. Thermal Evaporation Process

The bottom surface and walls of the channels were conformally coated with a 100 nm thick Cu film, deposited by a high-vacuum thermal evaporation technique at a pressure of 10^−6^ Torr. The chips were placed upside down in the chamber on a rotating holder at 45 RPM to ensure homogeneous coverage. A laser-cut Kapton mask, patterned using a 455 nm diode laser, defined the deposited area.

### 2.5. UV Window Sealing

To complete the device assembly, a transparent quartz window (2 cm × 2 cm, 500 µm thick) was placed on top of the chip to seal the channels and allow visualization of the flow and reaction inside the valve, as well as future optical analysis. The quartz window was fitted into the rectangular sealing frame of the chip, where it mechanically engaged with the printed structure. A minimal amount of photosensitive resin was placed over the available solid sealing area surrounding the microstructured region, while avoiding the channel itself, and was subsequently cured using a focused 405 nm UV laser. Under these conditions, the resin was not intentionally deposited over the internal C-shaped fish-scale microstructures. Therefore, a small initial gap may exist locally between the top of the fish-scales and the quartz window. However, in our experience, gaps of up to approximately 80 µm tend to close during the early stages of galvanic replacement due to the quasi-conformal Cu coating and the subsequent growth of metallic nanostructures from the coated surfaces.

### 2.6. Galvanic Replacement Reaction

After completing the procedures described in Section 2.5, we proceeded to synthesize Ag nanostructures via galvanic replacement using a microfluidic pump (Fluigent Flow EZ), which can operate over a pressure range of 1–1000 mbar with 1 mbar resolution. Nitrogen was used as the operating gas in all experiments. A 1 mM solution of AgNO_3_ was used as a precursor. The synthesis was conducted for 1 min at a constant flow rate of 10 µL/min. After this first experiment, the procedure was repeated using a HAuCl_4_ solution (1 mM) to synthesize Au nanostructures using another set of microreactors. A total of six microfluidic devices were successfully processed and used for galvanic replacement experiments (three devices for Ag and three devices for Au). Devices that showed leakage or unstable flow/pressure response were excluded prior to synthesis. All experiments were performed at room temperature and atmospheric pressure.

### 2.7. Replicates and Spatial Sampling

**Figure 2:**
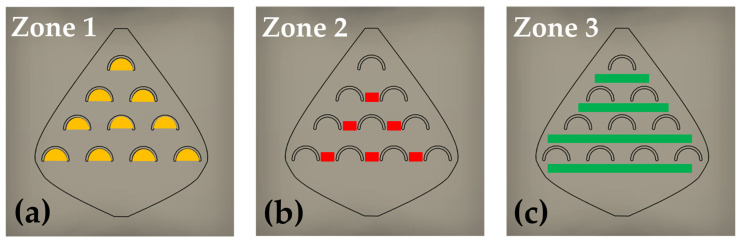
Schematic representation of the three zones of interest containing the three types of microregions: (**a**) inside the fish-scales (zone 1), (**b**) between the fish-scales (zone 2), and (**c**) along the rows of fish-scales (zone 3).

To characterize the spatial variability of the formed nanostructures, using the same device, the microreactors were divided into zones, each zone containing the same microregions, as shown in Figure 2. The repeated microregions are: inside fish-scales (zone 1), between the fish-scales (zone 2), and along the rows of fish-scales (zone 3). For each device, the number of available repeated regions (*n*) was: 1A: zone 1 (*n* = 10), zone 2 (*n* = 6), zone 3 (*n* = 4); 2A: zone 1 (*n* = 31), zone 2 (*n* = 26), zone 3 (*n* = 7); 3A: zone 1 (*n* = 91), zone 2 (*n* = 82), zone 3 (*n* = 11). For this count, incomplete fish-scales were excluded from zone 1, fish-scales located on the side walls were excluded from zone 2, and the row behind the last fish-scale was excluded from zone 3.

After synthesis, selected devices were mechanically opened to allow direct SEM observation of the nanostructures formed on the internal microchannel surfaces. Once opened, the devices were not considered for reassembly due to the increased risk of leakage.

SEM inspection and measurements were performed following a row-based sampling scheme. For each device, at least one SEM image was analyzed per row for zone 1, at least one SEM image per row for zone 2, and at least one SEM image per representative location along the rows for zone 3. Because each device design contains a different number of rows, the total number of analyzed SEM images per zone varied across 1A–3A. Within each analyzed SEM image, characteristic nanostructure dimensions were measured and recorded to capture the dimensional range observed within each zone.

### 2.8. Simulation Model

The pressure, velocity, shear-stress distribution, and *Re* in the studied devices were estimated by COMSOL Multiphysics software, version 5.4. The fish-scale valves used in the simulations were designed employing computer-aided design software, maintaining the same dimensions as the printed samples: a height of 200 µm, 16 mm long and 14 mm wide. As reported for structured microchannels, geometric features can strongly modify local hydrodynamic conditions and transport behavior, making numerical analysis a useful tool for evaluating the effect of design on flow-related phenomena [28].

The Reynolds number was obtained by:

(1) Re=ρul/μ

where *ρ* and *µ* are the density and dynamic viscosity of water, respectively (*ρ* = 1000 kg/m^3^ and *µ* = 0.001 Pa·s), *l* is the characteristic length (volume/area ratio) [29,30] of the channel shown in Table 1, and *u* is the fluid velocity along the pathway obtained by simulation. The motion of the fluid through the device was obtained by solving the Navier–Stokes equation using COMSOL:

(2) $$\rho \left(u\cdot \nabla u\right)=\nabla \cdot [-pl+\mu \left(\nabla u+{\left(\nabla u\right)}^{T}\right)]$$

and the continuity equation:

(3) ρ∇·(u)=0

For this study, we assume an incompressible flow in the device and simplify the Navier–Stokes equation by neglecting gravity. For maximum accuracy in the simulation, we generated 104,085; 366,944; and 594,742 tetrahedral elements for 1A, 2A, and 3A devices, respectively. At the inlet, we defined a fully developed flux velocity of 10 μL/min, equal to the one determined by the Fluigent Flow EZ micropump during the experiment. The boundary condition at the walls was set to *u* = 0, with no slip. The outlet of the devices was set as a static pressure point (*p* = 0).

## 3. Results and Discussion

### 3.1. Identification of Governing Flow Parameters

The fish-scale microchannel architecture defines three recurring microenvironments along the flow path in the three zones (Figure 2). The geometrical features impose zone-dependent hydrodynamic constraints at the Cu-coated surface: concave cavities promote low-velocity recirculation and localized vortices, whereas narrow gaps locally accelerate the flow and increase near-wall shear. Accordingly, the numerical model was used to extract velocity, shear stress, and Reynolds number fields to characterize the transport conditions within each zone.

**Figure 3:**
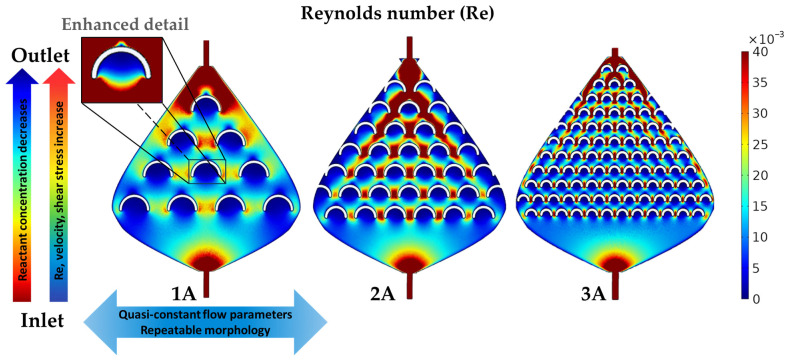
Reynolds number profiles for microreactor designs 1A, 2A, and 3A. The increasing *Re*, represented by a blue-to-red color scale, highlights well-defined zones of interest. The vertical arrows indicate variations in flow parameters and reactant concentration, while the horizontal arrow indicates quasi-static conditions across the microreactor width.

To relate nanostructure formation to the governing flow parameters, we selected zones of interest where NSs were predominantly observed throughout the device. The internal fish-scale microstructures promote localized vortices and recirculation whenever the flow interacts with the concave cavities, producing a heterogeneous distribution of velocities along the channel. Therefore, our analysis focuses on the regions where these geometry-induced perturbations may occur, primarily inside the fish-scales, between adjacent fish-scales, and along the edges of the valve. Numerical simulations show that the local *Re* remains very low throughout the device (*Re* ≤ 0.04 when using the scale spacing as the characteristic length). Inside the fish-scale cavities, *Re* decreases to the 10^−3^–10^−2^ range, resulting in a creeping-like, viscosity-dominated flow with steady geometry-induced recirculation as shown in Figure 3. In contrast, the regions between and along the scale rows exhibit slightly higher, yet still laminar, velocities (*Re* up to ≈0.04), consistent with accelerated flow through the narrow gaps between fish-scales.

**Figure 4:**
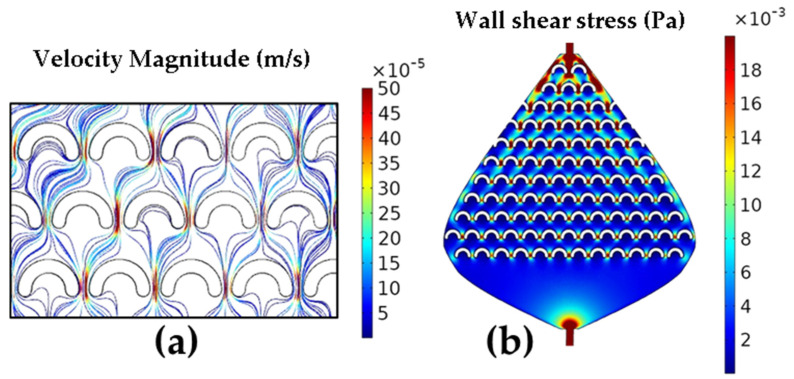
(**a**) Velocity magnitude and (**b**) Shear stress in the 3A microvalve shown in a blue-to-red scale.

**Figure 5:**
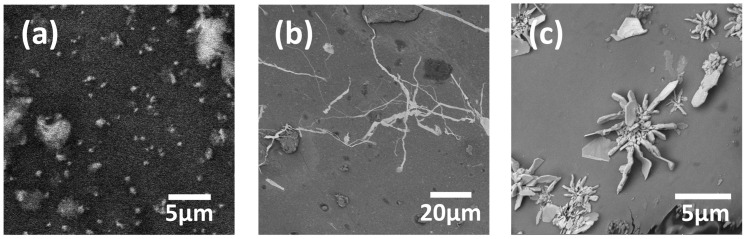
Ag nanostructures obtained within the microreactor channels: (**a**) Ag NPs with diameters ranging from 300 to 500 nm, formed in zone 1 of device 1A; (**b**) Ag NWs with widths between ≤100 and 300 nm, formed between the fish-scales in zone 2 of device 3A; (**c**) clusters of Ag NFs with a thickness of ~100 nm, formed along the first 3–4 rows of fish-scales in zone 3 near the microreactor inlet of device 2A.

**Figure 6:**
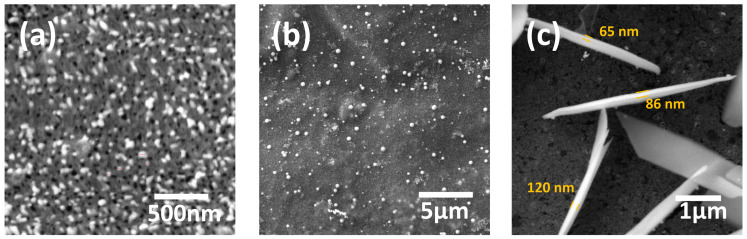
Au nanostructures obtained within the microvalve channels: (**a**) NPs (bright dots) and nanopores (black dots) with diameters ≤ 50 nm, formed in zone 1, in the first 4–5 lines close to the reactor inlet in device 1A; (**b**) Au NPs with diameters ranging from 100 to 450 nm, formed in zone 2 of device 3A; (**c**) Au nanowalls with a thickness of ~100 nm and a length of about 5 µm, formed near the inlet of device 3A.

In addition to the *Re*, the flow velocity and shear stress profiles were extracted from the simulation results. The shear stress was calculated as the product of the shear rate and the dynamic viscosity, where the shear rate was provided by the simulation. The streamlines shown in Figure 4a represent the flow velocity magnitude, with the fastest flow depicted by red lines between the fish-scales and the slowest flow depicted by blue lines inside the fish-scales, with intermediate flow velocities in the rest of the device areas. These three zones exhibit highly different velocity values, which match perfectly with the shear stress (Figure 4b) and *Re* results (Figure 3). Similarly, the nanostructures formed in these zones displayed a wide range of NS characteristics, as shown in Figure 5 and Figure 6 in the Experimental Verification section. The velocity magnitude, which depends on the flow rate, is important because of its relationship to the size and shape of the formed nanostructures [3] and because it can be directly controlled by the experimental setup. Similar simulation results were obtained for the flow in the three valves, with the most significant velocity increase between the fish-scales in valve 3A, where the fish-scales have the smallest lateral separation (x-spacing). The flow velocity is qualitatively related to nanostructure formation as a function of reaction time, with the red lines corresponding to the minimum possible reaction time (Figure 4a) and the blue lines indicating a longer reaction time.

Because these maps were obtained under a single (fixed) set of boundary conditions, spatial changes in velocity and shear stress arise primarily from the channel geometry rather than from varying the driving conditions from run to run. Therefore, the three-zone partition provides a consistent framework for comparing the local hydrodynamic forcing with the SEM-observed morphologies (Figure 5 and Figure 6) across 1A–3A.

Another variable considered is the wall shear stress. We plotted the shear stress along the channel surface (including walls and fish-scales), and a representative map for valve 3A is shown in Figure 4b. Shear stress is particularly relevant under the present low *Re* conditions, where viscous effects dominate near-wall transport and surface reactions.

The *Re* is related to the flow regime, which in turn influences how the precursor molecules interact with the surface of the nanostructures during galvanic displacement. Moreover, it is intrinsically related to other critical parameters, such as wall shear stress. As the *Re* increases, the wall shear stress also rises, as evidenced by the comparison of Figure 3 and Figure 4b [31]. This relationship is crucial for NS formation, influencing the distribution of sizes and shapes of NSs during the synthesis process. On the other hand, lower *Re* values inversely affect the residence time. Specifically, at low *Re*, the residence time increases, which explains the growth of larger NSs (Figure 5c and Figure 6c) due to a higher nucleation rate in regions of slow flow, as in the first rows of device 3A presented in Figure 2c.

A report on a microfluidic device used for the galvanic replacement of electrodeposited Cu in a solution of AgNO_3_ on a p-type Si substrate [32] shows that, at a low volumetric flow rate of 10 μL/min, as in the present study, Ag ions are deposited onto the substrate, resulting in the formation of spherical Ag nanoparticles. It also shows the correlation between wall shear stress and volumetric flow, which is a critical parameter in evaluating nanoparticle formation in a microfluidic device. Reports on the generation of metallic nanostructures in microfluidic devices [33] show that small increases in volumetric flow rate (4–8 μL/min) do not change the shape of the desired nanowall structures. However, a higher flow rate can lead to nanowalls with slightly larger dimensions due to a more efficient supply of reactants. In another report on the galvanic replacement of Cu nanostructures with a solution of AgNO_3_ in a microfluidic device [34], it was determined that the optimal concentration of AgNO_3_ at flow rate of 1 μL/min was 10 mM since at higher concentrations the mechanical strength of Ag-containing nanowalls is weak and the walls can be etched away. In the synthesis of Au-based nanostructures, there have been reports of reactions involving HAuCl_4_, H_3_PW_12_O_40_ (where PW is phosphotungstic acid anion), and H_3_PMo_12_O_40_ (where PMo is phosphomolybdate anion) in a microfluidic device [35], resulting in the formation of decorated Au@POM (where POM is a mixture of PW and PMo, called polyoxometalates) nanostructures within the microfluidic device at a volumetric flow rate of 1 mL/h for 2 h under constant UV irradiation.

At low velocities, mass transport toward the Cu surface becomes increasingly diffusion-limited, further enhancing nucleation in slow-flow regions. At low *Re* numbers, where inertial effects are negligible, the viscous forces of the fluid dominate, controlling the transport of precursor molecules and their deposition onto the surface. The *Re* varies along the reactors (Figure 3), reaching its maximum values between the fish-scales (zone 2) close to the outlet end of the valves. The minimum *Re* values were found near the first row of fish-scales at the inlet side, where the fluid velocity is lowest. This behavior is expected by design and is observed across all fish-scales and valves.

### 3.2. Experimental Verification

The formation of nanoparticles in the microchannels of fish valves initiates with the interaction of AgNO_3_ and HAuCl_4_ with the Cu film coating the microchannels. The size and shape of the resulting Ag and Au nanoparticles depend on reactant concentration [36], flow velocity [37], residence time [38,39], shear stress [40,41], and *Re* [42]. Due to the passage of reagents through the intricate patterns of the microvalves, the concentration of AgNO_3_ and HAuCl_4_ is reduced towards the outlet. In addition, the flow velocity, shear stress, and *Re* vary significantly along the flow paths. These variations are critical for the kinetics of nanostructure formation. Any nanostructure can change its shape or disappear when the balance between reagent concentrations and flow parameters is disrupted [43].

The design of the three types of reactors (Figure 2), with microstructures (fish-scales) arranged at strategic distances in order to modify fluid properties in a targeted manner, leads to the formation of various types of nanostructures (0D, 1D, and 2D) obtained in a laminar flow regime. The simulation results enabled the estimation of key parameters that cannot be directly measured, thereby allowing their correlation with nanostructures formed in specific zones. Three main zones within the microreactors were studied by SEM, those where the formed NS exhibited significant morphological differences: inside the fish-scales, between the fish-scales, and along the rows of fish-scales (Figure 2).

#### 3.2.1. Ag Nanostructures

The process occurring inside the microfluidic device is governed by a heterogeneous galvanic displacement reaction between Ag^+^ ions in the 10 mM AgNO_3_ solution and the Cu thin film coating the internal surfaces. In this reaction, Cu is oxidized to Cu^2+^, releasing electrons that immediately reduce Ag^+^ to metallic Ag on the Cu surface, forming nanostructures across the film. Because this interfacial mechanism is strongly influenced by local hydrodynamics, the number and spatial arrangement of the fish-scale features introduce regions with distinct flow characteristics, residence times, and shear conditions. Each of these regions functions effectively as a microscale flow valve, creating locally different synthesis conditions within the same device. Incorporating multiple fish-scales, therefore, enables controlled modulation of nanostructure growth and allows systematic evaluation of how flow parameters govern nanostructure morphology and distribution.

In the three microreactors 1A, 2A, and 3A, semispherical nanoparticles (NPs) with diameters in the 200–500 nm range (Figure 5a) were observed in zone 1. Inside the fish-scales, the smallest values of *Re*, shear stress, and flow velocity were obtained in all fish-scale valve designs, allowing nucleation of particles in a steady-like flow. Additionally, some kinds of vortices were detected in this zone, hindering the replenishment of the scale cavities and limiting the reagent concentration and the galvanic replacement reaction. Figure 3 shows a detailed image of a scale with enhanced contrast, highlighting the *Re* distribution inside the cavity.

In the regions between the fish-scales (zone 2), depicted as red lines (Figure 2b), nanowires (NWs) with widths ranging from ≈100 nm to 900 nm and lengths of 20–100 µm were formed (Figure 5b), primarily in device 3A and less frequently in device 2A. As determined by the simulation, the *Re*, shear stress, and flow velocity between the fish-scales reached their highest values, leading to the growth of elongated nanostructures as they were dragged in the flow direction. In device 2A, clusters of nanoflakes (NFs) were formed in the regions along the first 3–4 green lines (zone 3) near the microreactor inlet, where the AgNO_3_ concentration was sufficient. The NFs had a thickness of ~100 nm and a length between 1 and 3 µm (Figure 5c). The *Re*, shear stress, and flow velocity in zone 3 were intermediate compared to the previous two zones in all Ag and Au experiments.

#### 3.2.2. Au Nanostructures

The flow parameters determined using COMSOL for the HAuCl_4_ experiments were the same as for the Ag experiments in all zones and microreactor designs. In zone 1, within the first 4–5 rows near the reactor inlet, NPs (bright dots) and nanopores (black dots) were observed in all three microreactor designs (Figure 6a). The classical redox reaction leads to the formation of Au NPs with an average size of 50 nm and nanopores with a diameter ≤ 50 nm, driven by the reduction of Au and oxidation of Cu [44,45]. The nanopores are formed by corrosion due to the HCl byproduct, which decreases the pH of the solution [46]. Nanopores were also observed in zones 2 and 3; however, compared with zone 1, they appear less frequently, with larger and more irregular features. In zone 2, Au NPs with diameters of 100 to 450 nm were observed in all microreactors (Figure 6b). The formation of larger diameter NPs is attributed to the replenishment of HAuCl_4_ compared to zone 1. In zone 3, nanowalls with a thickness of ~100 nm and a length of about 5 µm were formed in the microreactor 3A (Figure 6c). The thickness was obtained when measured at the thinnest visible edges of the nanowalls. The transition from Au nanoparticles to nanowalls is consistent with zone-dependent local reaction conditions at the Cu-coated surface created by the valve architecture. Near the inlet, the precursor concentration is highest, and the local hydrodynamics include low-velocity recirculation and longer residence-time regions, which increase the local availability of Au species at the Cu-coated surface and promote coalescence and anisotropic growth into extended 2D wall-like features. Downstream and outside this localized region, the reactant concentration decreases along the reactor, and the local flow is more advective and shear-influenced in the narrow gaps, which favors repeated nucleation and the formation of discrete semispherical nanoparticles. This interpretation links the observed morphologies to the zone-resolved hydrodynamic fields obtained from the simulations. The nanowalls were observed only in the 3A microreactor at points where the minimum flow velocity values and the maximum residence time were obtained, due to flow resistance between fish-scales. The maximum quantity of nanowalls was found along the first row of fish-scales, where the HAuCL_4_ concentration was enough to sustain the reaction. The quantity of nanowalls rapidly decreases and then becomes zero in the direction of the outlet, where the HAuCl_4_ concentration decreases and the flow velocity increases.

#### 3.2.3. Stability and Zone-Dependent Morphology

The stability of the proposed platform was assessed at the device and spatial levels. At the device level, operational robustness was ensured by pre-screening the valves for continuous flow and leak-free operation prior to synthesis, so that galvanic replacement was performed under stable nominal conditions. In addition, channel cleaning under increased flow conditions showed that the formed material can be detached, indicating that flushing-based removal is possible if reuse of the same geometry is desired for testing different operating conditions or for ex situ characterization of the nanostructures. At the spatial level, stability refers to the repeatability of zone-dependent outcomes within a given architecture: across the inspected SEM regions, the dominant morphology recurred within each predefined zone across the device width, while variability was primarily reflected in characteristic size and areal density along the inlet-to-outlet direction, as summarized in Figure 3. A zone-resolved summary is provided in Table 2. These observations support the role of the fish-scale architecture in defining local microenvironments, such as concave cavities that promote low-velocity recirculation and localized vortices versus narrow gaps that locally accelerate the flow and increase the shear stress near the wall, which bias nucleation and growth pathways. Moreover, the architectural complexity provides repeated microregions within a single valve, enabling multiple internal comparisons within the same run and reducing run-to-run variability arising from handling and setup. Consequently, the valve design enables systematic spatial modulation of nanostructure morphology (0D/1D/2D) as a function of zone and geometry under the selected flow conditions, despite variability in characteristic size and areal density.

In addition, these zone-dependent morphologies suggest practical routes for tailoring plasmonic surfaces within a single microfluidic platform. Nanoparticle and nanopore-rich regions can increase the density of curvature- and gap-driven electromagnetic hot spots, while wire-like and wall-like morphologies provide extended edges and junction networks that can further intensify localized fields. Therefore, the proposed architecture enables low-cost screening and spatial selection of plasmonic morphologies commonly associated with SERS substrates and biosensors, without requiring electrochemical processing.

## 4. Conclusions

This study successfully demonstrates the use of 3D-printed microfluidic devices for flow-driven surface modification and the synthesis of Ag and Au nanostructures via galvanic displacement, depending on microregion conditions. The unique internal geometries of the microreactors, characterized by C-shaped scale-like microstructures, allowed for the controlled formation of various nanostructures like nanoparticles, nanowires, nanoflakes, and nanowalls, through the modulation of flow parameters such as velocity, shear stress, and Reynolds number. Moreover, a strong relation was demonstrated between physical and chemical parameters (flow velocity and concentration gradient) and the nanostructure formation. Across the valve width, the dominant morphology in each zone was highly repeatable as confirmed by SEM, and variations were mainly observed along the flow direction from inlet to outlet. Lateral edge regions can deviate from the central pattern due to the intentionally asymmetric inlet-to-outlet design and local fabrication tolerances, which reduce symmetry. This spatial behavior is consistent with the zone-resolved hydrodynamic conditions summarized by the KPIs. The COMSOL simulations effectively correlated the flow characteristics with the observed nanostructure morphologies. The findings highlight the potential of these microfluidic systems to fine-tune the synthesis process toward applications such as surface enhanced Raman spectroscopy (SERS) and rapid prototyping of NP-based biosensors, without the need for electrochemical reactions. This approach supports low-cost, spatially resolved control of nanostructure morphology in microfluidic environments and provides a basis to design simplified valves that reproduce the determining local conditions to selectively obtain a target morphology.

## Figures and Tables

**Table 1:** Dimensions of characteristic features in the microfluidic fish-scale valve devices 1A, 2A, and 3A.

Dimension/Label	1A	2A	3A
Fish-scale quantity	10	33	100
Height (µm)	200	200	200
Width (µm)	200	200	200
Radius (µm)	900	600	250
x-spacing (µm)	1300	500	150
y-spacing (µm)	1300	600	450
Characteristic length l (µm)	91	84	77

**Table 2:** Zone-resolved *Re* ranges and characteristic dimensions of Ag and Au nanostructures.

Device/Zone	*Re* Range	Ag	Characteristic Dimensions	Au	Characteristic Dimensions
A1/Z1	8.28 × 10^−4^–6.64 × 10^−3^	NPs	324 ± 94 nm (n = 50)	NPs, Nanopores	35 ± 6 nm (n = 60)
A1/Z2	3.29 × 10^−2^–5.58 × 10^−2^	NPs	324 ± 94 nm (n = 50)	NPs, Nanopores	221 ± 101 nm (n = 24)
A1/Z3	8.02 × 10^−3^–4.94 × 10^−2^	NPs	324 ± 94 nm (n = 50)	NPs	221 ± 101 nm (n = 24)
A2/Z1	1.07 × 10^−2^–3.60 × 10^−2^	NPs	324 ± 94 nm (n = 50)	NPs, Nanopores	35 ± 6 nm (n = 60)
A2/Z2	1.08 × 10^−1^–1.82 × 10^−1^	NWs	567 ± 422 nm (n = 26)	NPs	221 ± 101 nm (n = 24)
A2/Z3	9.34 × 10^−2^–1.10 × 10^−1^	NFs	184 ± 42 nm (n = 24)	Nanowalls	107 ± 35 nm (n = 20)
A3/Z1	5.87 × 10^−4^–1.33 × 10^−2^	NPs	324 ± 94 nm (n = 50)	NPs, Nanopores	35 ± 6 nm (n = 60)
A3/Z2	5.46 × 10^−2^–2.04 × 10^−1^	NWs	567 ± 422 nm (n = 26)	NPs	221 ± 101 nm (n = 24)
A3/Z3	1.06 × 10^−2^–1.43 × 10^−1^	NPs	324 ± 94 nm (n = 50)	NPs	221 ± 101 nm (n = 24)

## Data Availability

The original contributions presented in this study are included in the article. Further inquiries can be directed to the corresponding author.
